# Supplementary material for: Health Risk Exposure Assessment of Migration of Perfluorooctane Sulfonate and Perfluorooctanoic Acid from Paper and Cardboard in Contact with Food under Temperature Variations
Source: Foods. 2023 Apr 24;12(9):1764. doi: 10.3390/foods12091764 (PMC10178734; doi:10.3390/foods12091764)
Supplement: Supplementary file 1 [file foods-12-01764-s001.zip › foods-2297552-supplementary.pdf]

Table S1.

Reference	PFOS			PFOA			Instrument
	**LOD (ng g^{-1})**	**LOQ (ng g^{-1})**	**Recovery±SD (%)**	**LOD (ng g^{-1})**	**LOQ (ng g^{-1})**	**Recovery±SD (%)**	
Sinclair *et al*., 2007	-	-	-	N/A	N/A	100±30	LC-MS/MS
Dolman and Pelzing, 2011	-	-	-	25 pg mL^{-1}	50 pg mL^{-1}	81.5-88.7	LC-MS
Martínez-Moral *et al*., 2012	2.2	5.0	108±21[a]	18	53	108±15[a]	LC-QTOF-MS/MS
Poothong *et al*., 2012	N/A	N/A	N/A	N/A	N/A	N/A	LC-MS/MS
Moreta and Tena, 2013	0.9	3.0	96-108[a]	2.2	7.0	96-104[a]	LC-QTOF-MS/MS
Zafeiraki *et al*., 2014	0.49	1.48	60-90	0.6	1.82	60-90	LC-MS/MS
Surma *et al*., 2015	0.04 pg cm^{-2}	0.1 pg cm^{-2}	91±7.2	0.01 pg cm^{-2}	0.03 pg cm^{-2}	89±6.3	LC-MS/MS
Shoeib *et al*., 2016	N/A	N/A	51.2±7.2	N/A	N/A	80.4±14	LC-MS/MS
Yuan *et al*., 2016	-	-	-	N/A	0.1	91±3	LC-QTOF-MS/MS
Zabaleta *et al*., 2017	-	-	-	1.9	N/A	95±14	LC-QTOF-MS

Abbreviations: Limit of detection (LOD); Limit of quantitation (LOQ); Standard deviation (SD); Not available (N/A); Liquid chromatography-quadrupole-time of flight mass spectrometer (LC-QTOF-MS/MS).
- Not analyzed in the study.
[a] Recovery±95% confidence interval.

Table S2.

Food contact paper and board	PFOS concentration	Converted concentration (ng cm^{-2})	Reference
Popcorn bag	<LOQ[a]	0.0200	Martínez-Moral *et al.*, 2012
	12 ng g^{-1}	0.0960	
	23 ng g^{-1}	0.1840	
	2.54 ng dm^{-2}	0.0254	Poothong *et al.*, 2012
	2.48 ng dm^{-2}	0.0248	
	ND[b]	0	
	2.26 ng dm^{-2}	0.0226	
	5.9 ng g^{-1}	0.0472	Moreta and Tena, 2013
	7.7 ng g^{-1}	0.0616	
	<LOQ[a]	0.0120	
	<LOQ[a]	0.0120	
	ND[c]	0.0036	
	ND[c]	0.0036	
	5.0 ng g^{-1}	0.0400	
	5.4 ng g^{-1}	0.0432	
	4.6 ng g^{-1}	0.0368	
	5.6 ng g^{-1}	0.0448	
	<LOQ[a]	0.0120	
	<LOQ[a]	0.0120	
	<LOD[c]	0.0020	Zafeiraki *et al.*, 2014
Paper tableware	7.74 ng dm^{-2}	0.0774	Poothong *et al.*, 2012
	8.48 ng dm^{-2}	0.0848	
	7.22 ng dm^{-2}	0.0722	
	8.72 ng dm^{-2}	0.0872	
	8.25 ng dm^{-2}	0.0825	
	ND[b]	0	
	7.37 ng dm^{-2}	0.0737	
	7.44 ng dm^{-2}	0.0744	
	7.53 ng dm^{-2}	0.0753	
	7.44 ng dm^{-2}	0.0744	
	9.83 ng dm^{-2}	0.0983	
	ND[b]	0	
Paper box	12.56 ng dm^{-2}	0.1256	Poothong *et al.*, 2012
	9.15 ng dm^{-2}	0.0915	
	92.48 ng dm^{-2}	0.9248	
	7.52 ng dm^{-2}	0.0752	
	6.90 ng dm^{-2}	0.0690	
	6.02 ng dm^{-2}	0.0602	
	8.79 ng dm^{-2}	0.0879	
	8.60 ng dm^{-2}	0.0860	
	8.25 ng dm^{-2}	0.0825	
	8.40 ng dm^{-2}	0.0840	

Table S2. (continued)

Food contact paper and board	PFOS concentration	Converted concentration (ng cm^{-2})	Reference
Paper box	10.04 ng dm^{-2}	0.1004	Poothong *et al.*, 2012
	9.92 ng dm^{-2}	0.0992	
	<LOD[c]	0.0063	Zafeiraki *et al.*, 2014
	0.18 ng g^{-1}	0.0046	Shoeib *et al.*, 2016
	0.13 ng g^{-1}	0.0034	
	3.82 ng g^{-1}	0.0985	
Paper cup	11.45 ng dm^{-2}	0.1145	Poothong *et al.*, 2012
	7.44 ng dm^{-2}	0.0744	
	9.62 ng dm^{-2}	0.0962	
	10.26 ng dm^{-2}	0.1026	
	8.43 ng dm^{-2}	0.0843	
	ND[b]	0	
	5.7 ng g^{-1}	0.1629	Moreta and Tena, 2013
	7.2 ng g^{-1}	0.2057	
	<LOD[c]	0.0070	Zafeiraki *et al.*, 2014
Paper bag	8.60 ng dm^{-2}	0.0860	Poothong *et al.*, 2012
	2.23 ng dm^{-2}	0.0223	
	1.37 ng dm^{-2}	0.0137	
	1.45 ng dm^{-2}	0.0145	
	ND[b]	0	
	1.42 ng dm^{-2}	0.0142	
	ND[c]	0	Surma *et al.*, 2015
	ND[c]	0	
	ND[c]	0	
	1.81 pg cm^{-2}	0.0018	
	5.17 pg cm^{-2}	0.0052	
	1.38 pg cm^{-2}	0.0014	
Wrapper	9.16 ng dm^{-2}	0.0916	Poothong *et al.*, 2012
	1.24 ng dm^{-2}	0.0124	
	2.94 ng dm^{-2}	0.0294	
	1.41 ng dm^{-2}	0.0141	
	0.86 ng dm^{-2}	0.0086	
	0.86 ng dm^{-2}	0.0086	
	1.40 ng dm^{-2}	0.0140	
	ND[b]	0	
	0.66 ng dm^{-2}	0.0066	
	ND[b]	0	
	ND[b]	0	
	ND[b]	0	
	1.33 ng dm^{-2}	0.0133	
	ND[b]	0	
	<LOD[c]	0.0007	Zafeiraki *et al.*, 2014

Table S2. (continued)

Food contact paper and board	PFOS concentration	Converted concentration (ng cm^{-2})	Reference
Wrapper	<LOD[c]	0.0007	Zafeiraki *et al.*, 2014
	ND[c]	0	Surma *et al.*, 2015
	ND[c]	0	
	ND[c]	0	
	ND[c]	0	
	ND[c]	0	
	ND[c]	0	
	4.90 ng g^{-1}	0.0150	Shoeib *et al.*, 2016
	0.33 ng g^{-1}	0.0010	
	0.34 ng g^{-1}	0.0010	
	0.33 ng g^{-1}	0.0010	
	0.38 ng g^{-1}	0.0012	
	0.16 ng g^{-1}	0.0005	
	0.25 ng g^{-1}	0.0008	
	0.11 ng g^{-1}	0.0003	
	0.50 ng g^{-1}	0.0015	
	0.04 ng g^{-1}	0.0001	
	0.12 ng g^{-1}	0.0004	

Abbreviations: Limit of quantification (LOQ); Non-detection (ND); Limit of detection (LOD).

[a] Below LOQ values were set to 1/2 LOQ.

[b] If without LOD value, ND were set to 0.

[c] ND and below LOD values were set to 1/2 LOD.

Table S3.

Food contact paper and board	PFOA concentration	Converted concentration (ng cm^{-2})	Reference
Popcorn bag	4.7 ng cm^{-2}	4.7000	Sinclair *et al.*, 2007
	0.3 ng cm^{-2}	0.3000	
	9.1 μg kg^{-1}	0.0728	Dolman and Pelzing, 2011
	<LOQ[a]	0.0004	
	53 ng g^{-1}	0.4240	Martínez-Moral *et al.*, 2012
	88 ng g^{-1}	0.7040	
	198 ng g^{-1}	1.5840	
	1.71 ng dm^{-2}	0.0171	Poothong *et al.*, 2012
	1.44 ng dm^{-2}	0.0144	
	0.12 ng dm^{-2}	0.0012	
	2.90 ng dm^{-2}	0.0290	
	ND[c]	0.0088	Moreta and Tena, 2013
	ND[c]	0.0088	
	14 ng g^{-1}	0.1120	
	15 ng g^{-1}	0.1200	
	7.2 ng g^{-1}	0.0576	
	<LOQ[a]	0.0280	
	ND[c]	0.0088	
	ND[c]	0.0088	
	ND[c]	0.0088	
	ND[c]	0.0088	
	ND[c]	0.0088	
	ND[c]	0.0088	
	<LOD[c]	0.0024	Zafeiraki *et al.*, 2014
	45.6 ng g^{-1}	0.3648	Yuan *et al.*, 2016
	27 ng g^{-1}	0.2160	Zabaleta *et al.*, 2017
	4.6 ng g^{-1}	0.0368	
	22 ng g^{-1}	0.1760	
	4.4 ng g^{-1}	0.0352	
	<LOD[c]	0.0076	
	<LOD[c]	0.0076	
	<LOD[c]	0.0076	
	3.9 ng g^{-1}	0.0312	
	4.2 ng g^{-1}	0.0336	
	4.5 ng g^{-1}	0.0360	
	3.6 ng g^{-1}	0.0288	
	<LOD[c]	0.0076	
	<LOD[c]	0.0076	
	4.1 ng g^{-1}	0.0328	
	ND[c]	0.0076	
	4 ng g^{-1}	0.0320	
	3.7 ng g^{-1}	0.0296	

Table S3. (continued)

Food contact paper and board	PFOA concentration	Converted concentration ($ng\ cm^{-2}$)	Reference
Popcorn bag	<LOD[c]	0.0076	Zabaleta *et al.*, 2017
	3 $ng\ g^{-1}$	0.0240	
	ND[c]	0.0076	
	<LOD[c]	0.0076	
	<LOD[c]	0.0076	
	<LOD[c]	0.0076	
	<LOD[c]	0.0076	
	<LOD[c]	0.0076	
	<LOD[c]	0.0076	
	<LOD[c]	0.0076	
	ND[c]	0.0076	
	3.5 $ng\ g^{-1}$	0.0280	
	3.8 $ng\ g^{-1}$	0.0304	
	<LOD[c]	0.0076	
	<LOD[c]	0.0076	
	<LOD[c]	0.0076	
	<LOD[c]	0.0076	
	<LOD[c]	0.0076	
	<LOD[c]	0.0076	
	<LOD[c]	0.0076	
	<LOD[c]	0.0076	
	<LOD[c]	0.0076	
	ND[c]	0.0076	
	<LOD[c]	0.0076	
	51 $ng\ g^{-1}$	0.4080	
	56 $ng\ g^{-1}$	0.4480	
Paper tableware	8.82 $ng\ dm^{-2}$	0.0882	Poothong *et al.*, 2012
	4.95 $ng\ dm^{-2}$	0.0495	
	4.21 $ng\ dm^{-2}$	0.0421	
	1.10 $ng\ dm^{-2}$	0.0110	
	9.55 $ng\ dm^{-2}$	0.0955	
	ND[b]	0	
	6.11 $ng\ dm^{-2}$	0.0611	
	4.17 $ng\ dm^{-2}$	0.0417	
	2.36 $ng\ dm^{-2}$	0.0236	
	3.44 $ng\ dm^{-2}$	0.0344	
	6.75 $ng\ dm^{-2}$	0.0675	
	ND[b]	0	
	31.1 $ng\ g^{-1}$	0.8663	Yuan *et al.*, 2016
Paper box	ND[c]	0.0032	Dolman and Pelzing, 2011
	16.07 $ng\ dm^{-2}$	0.1607	Poothong *et al.*, 2012
	15.15 $ng\ dm^{-2}$	0.1515	

Table S3. (continued)

Food contact paper and board	PFOA concentration	Converted concentration (ng cm^{-2})	Reference
Paper box	3.86 ng dm^{-2}	0.3860	Poothong *et al.*, 2012
	17.74 ng dm^{-2}	0.1774	
	5.79 ng dm^{-2}	0.5790	
	5.69 ng dm^{-2}	0.5690	
	1.16 ng dm^{-2}	0.1160	
	1.86 ng dm^{-2}	0.1860	
	1.15 ng dm^{-2}	0.1150	
	12.34 ng dm^{-2}	0.1234	
	<LOD[c]	0.0077	Zafeiraki *et al.*, 2014
	7.53 ng g^{-1}	0.1941	Shoeib *et al.*, 2016
	0.62 ng g^{-1}	0.0160	
	2.35 ng g^{-1}	0.0606	
	0.54 ng g^{-1}	0.0139	
	0.57 ng g^{-1}	0.0147	Yuan *et al.*, 2016
Paper cup	8.11 ng dm^{-2}	0.0811	Poothong *et al.*, 2012
	1.51 ng dm^{-2}	0.0151	
	3.01 ng dm^{-2}	0.0301	
	2.06 ng dm^{-2}	0.0206	
	10.29 ng dm^{-2}	0.0103	
	ND[b]	0	
	ND[c]	0.0314	Moreta and Tena, 2013
	<LOD[c]	0.0086	Zafeiraki *et al.*, 2014
	3.63 ng g^{-1}	0.1037	Yuan *et al.*, 2016
Paper bag	4.36 ng dm^{-2}	0.0436	Poothong *et al.*, 2012
	3.28 ng dm^{-2}	0.0328	
	0.65 ng dm^{-2}	0.0065	
	2.66 ng dm^{-2}	0.0266	
	0.9 ng dm^{-2}	0.0090	
	0.79 ng dm^{-2}	0.0079	
	2.06 pg cm^{-2}	0.0206	Surma *et al.*, 2015
	6.22 pg cm^{-2}	0.0062	
	5.00 pg cm^{-2}	0.0050	
	0.10 pg cm^{-2}	0.0001	
	0.22 pg cm^{-2}	0.0002	
	0.13 pg cm^{-2}	0.0001	
	1.07 ng g^{-1}	0.0050	Shoeib *et al.*, 2016
	1.07 ng g^{-1}	0.0050	

Table S3. (continued)

Food contact paper and board	PFOA concentration	Converted concentration (ng cm^{-2})	Reference
Paper bag	0.57 ng g^{-1}	0.0027	Shoeib *et al.*, 2016
	0.77 ng g^{-1}	0.0036	
	0.26 ng g^{-1}	0.0012	Yuan *et al.*, 2016
Wrapper	ND[c]	0.0004	Dolman and Pelzing, 2011
	ND[c]	0.0004	
	ND[c]	0.0004	
	4.16 ng dm^{-2}	0.0416	Poothong *et al.*, 2012
	3.72 ng dm^{-2}	0.0372	
	2.36 ng dm^{-2}	0.0236	
	1.77 ng dm^{-2}	0.0177	
	0.42 ng dm^{-2}	0.0042	
	0.99 ng dm^{-2}	0.0099	
	0.52 ng dm^{-2}	0.0052	
	0.72 ng dm^{-2}	0.0072	
	0.41 ng dm^{-2}	0.0041	
	ND[b]	0	
	0.09 ng dm^{-2}	0.0009	
	0.34 ng dm^{-2}	0.0034	
	1.23 ng dm^{-2}	0.0123	
	ND[b]	0	
	<LOD[c]	0.0009	Zafeiraki *et al.*, 2014
	<LOD[c]	0.0009	
	1.30 pg cm^{-2}	0.0013	Surma *et al.*, 2015
	2.22 pg cm^{-2}	0.0022	
	2.49 pg cm^{-2}	0.0025	
	0.85 pg cm^{-2}	0.0009	
	1.57 pg cm^{-2}	0.0016	
	1.39 pg cm^{-2}	0.0014	
	65.33 ng g^{-1}	0.1997	Shoeib *et al.*, 2016
	5.12 ng g^{-1}	0.0156	
	11.73 ng g^{-1}	0.0358	
	8.81 ng g^{-1}	0.0269	
	3.48 ng g^{-1}	0.0106	
	4.00 ng g^{-1}	0.0122	
	3.72 ng g^{-1}	0.0114	
	1.38 ng g^{-1}	0.0042	

Table S3. (continued)

**Food contact paper and board**	**PFOA concentration**	**Converted concentration ($ng\ cm^{-2}$)**	**Reference**
Wrapper	93.62 $ng\ g^{-1}$	0.2861	Shoeib *et al.*, 2016
	2.40 $ng\ g^{-1}$	0.0073	

Abbreviations: Limit of quantification (LOQ); Non-detection (ND); Limit of detection (LOD).

[a] Below LOQ values were set to 1/2 LOQ.

[b] If without LOD value, ND were set to 0.

[c] ND and below LOD values were set to 1/2 LOD.

Table S4.

	Percent of dose							
	Rat		Mouse		Hamster		Rabbit	
Sample (%)	Male	Female	Male	Female	Male	Female	Male	Female
Urine	25.6	73.9	3.4	6.7	90.3	45.3	76.8	87.9
Tissues	59.6	0.6	73.6	50	0.7	26.5	<0.1	0.3
Expiration products	3.5	1.5	5.2	4.4	1.3	2.9	-	-
Cage wash	0.6	0.8	4.9	4.9	0.6	2.1	0.5	4.8
Recovered	89.3	76.8	87.1	66	92.9	76.8	77.3	93

Table S5.

	**Male (n = 30)**	**Female (n = 29)**	**Total (n = 59)**
PFOS			
Range	4.67 – 22.61*	3.45 – 25.65*	3.45 – 25.65*
Median	10.23	6.01	8.52
GM(SD)	10.64 (5.10)	7.12 (5.61)	8.96 (5.67)
PFOA			
Range	2.07 – 7.69*	1.55 – 5.98*	1.55 – 7.69*
Median	3.77	2.48	3.22
GM(SD)	3.83 (1.24)	2.77 (1.21)	3.27 (1.32)

Abbreviations: Geometric mean (GM); Standard deviation(SD).

* Minimum – Maximum.

Table S6.

Quantile	Median PFOS ($ng\ mL^{-1}$)	PFOS intake* ($ng\ kg\text{-}bw^{-1}\ day^{-1}$)	Total cholesterol concentration ($mg\ dL^{-1}$)	Reference
1	9.9	1.0	200	Nelson *et al.*, 2009
2	17	1.8	205.9	
3	24	2.5	205	
4	38	4.0	213.6	
1	6.4	0.7	197	Steenland *et al.*, 2009
2	10.5	1.1	199.3	
3	13.6	1.4	200	
4	16.1	1.7	203	
5	18.8	2.0	204	
6	21.6	2.3	205	
7	24.9	2.6	205.7	
8	29.2	3.1	207.7	
9	35.5	3.7	209.2	
10	49.3	5.2	209.6	
1	17	1.8	223	Eriksen *et al.*, 2013
2	23.7	2.5	230.3	
3	28.4	3.0	235.6	
4	32.2	3.4	233	
5	36.9	3.9	235	
6	41.1	4.3	237.5	
7	47.9	5.1	239	
8	58.5	6.2	234	

* Converted by $DP = CP \times KP \times Vd$.

DP: Dietary intakes of PFOS and PFOA ($ng\ kg\text{-}bw^{-1}\ day^{-1}$).

CP: PFOS and PFOA concentrations in serum or plasma ($ng\ mL^{-1}$).

KP: Elimination rates of PFOS and PFOA (day^{-1}).

Vd: Distribution volumes of PFOS and PFOA ($mL\ kg\text{-}bw^{-1}$).

Table S7.

Quantile	Median PFOA ($ng\ mL^{-1}$)	PFOA intake* ($ng\ kg\text{-}bw^{-1}\ day^{-1}$)	Total cholesterol concentration ($mg\ dL^{-1}$)	Reference
1	5.5	1.4	199	Steenland *et al.*, 2009
2	9.6	2.4	202	
3	13.5	3.3	204	
4	18.2	4.5	205	
5	24.1	6.0	206	
6	33.5	8.3	206	
7	48.3	12.0	208	
8	70.9	17.6	207	
1	3.3	0.8	223	Eriksen *et al.*, 2013
2	4.6	1.1	226.5	
3	5.4	1.3	231.5	
4	6.2	1.5	229	
5	7.1	1.8	226.2	
6	7.9	2.0	237.5	
7	9.3	2.3	229.4	
8	11.8	2.9	235.4	

* Converted by $DP = CP \times KP \times Vd$.

DP: Dietary intakes of PFOS and PFOA ($ng\ kg\text{-}bw^{-1}\ day^{-1}$).

CP: PFOS and PFOA concentrations in serum or plasma ($ng\ mL^{-1}$).

KP: Elimination rates of PFOS and PFOA (day^{-1}).

Vd: Distribution volumes of PFOS and PFOA ($mL\ kg\text{-}bw^{-1}$).

Table S8.

Compound	Half-life (years)	Reference	Distribution volume (mL kg-bw^{-1})	Reference
PFOS	5.4	Olsen *et al*., 2007	300	Harada *et al*., 2005
PFOA	2.3	Bartell *et al*., 2009	300	Harada *et al*., 2005

Table S9.

Uptake dose (%-tile)	High-exposure scenario				Intermediate-exposure scenario				Low-exposure scenario			
	Male		Female		Male		Female		Male		Female	
	HT	LT	HT	LT	HT	LT	HT	LT	HT	LT	HT	LT
**PFOS**												
**100**	2.1×10^{0}	1.1×10^{0}	2.2×10^{0}	1.2×10^{0}	1.6×10^{-1}	8.0×10^{-2}	1.6×10^{-1}	9.0×10^{-2}	1.6×10^{-3}	8.4×10^{-4}	1.6×10^{-3}	8.6×10^{-4}
**95**	3.2×10^{-1}	1.7×10^{-1}	3.7×10^{-1}	2.0×10^{-1}	2.4×10^{-2}	1.3×10^{-2}	2.8×10^{-2}	1.5×10^{-2}	2.4×10^{-4}	1.3×10^{-4}	2.8×10^{-4}	1.5×10^{-4}
**50**	1.7×10^{-2}	9.3×10^{-3}	2.0×10^{-2}	1.1×10^{-2}	1.3×10^{-3}	7.0×10^{-4}	1.6×10^{-3}	8.4×10^{-4}	1.0×10^{-5}	1.0×10^{-5}	2.0×10^{-5}	1.0×10^{-5}
**5**	7.8×10^{-4}	4.2×10^{-4}	9.4×10^{-4}	5.0×10^{-4}	6.0×10^{-5}	3.0×10^{-5}	7.0×10^{-5}	4.0×10^{-5}	5.8×10^{-7}	3.1×10^{-7}	7.0×10^{-7}	3.7×10^{-7}
**PFOA**												
**100**	7.9×10^{0}	4.2×10^{0}	7.7×10^{0}	4.1×10^{0}	6.0×10^{-1}	3.2×10^{-1}	5.8×10^{-1}	3.1×10^{-1}	6.0×10^{-3}	3.2×10^{-3}	6.0×10^{-3}	3.1×10^{-3}
**95**	3.4×10^{-1}	1.8×10^{-1}	4.1×10^{-1}	2.2×10^{-1}	2.6×10^{-2}	1.4×10^{-2}	3.0×10^{-2}	1.6×10^{-2}	2.6×10^{-4}	1.4×10^{-4}	3.1×10^{-4}	1.6×10^{-4}
**50**	1.6×10^{-2}	8.3×10^{-3}	1.8×10^{-2}	9.8×10^{-3}	1.2×10^{-3}	6.2×10^{-4}	1.4×10^{-3}	7.3×10^{-4}	1.0×10^{-5}	1.0×10^{-5}	1.0×10^{-5}	1.0×10^{-5}
**5**	6.2×10^{-4}	3.3×10^{-4}	7.5×10^{-4}	4.0×10^{-4}	5.0×10^{-5}	2.0×10^{-5}	6.0×10^{-5}	3.0×10^{-5}	4.7×10^{-7}	2.5×10^{-7}	5.6×10^{-7}	3.0×10^{-7}

Abbreviations: High temperature (HT); Low temperature (LT).

Table S10.

	Male (n = 30)*	Female (n = 29)*	Total (n = 59)*
PFOS			
Maximum	2.39	2.71	2.71
Minimum	0.49	0.36	0.36
Median	1.08	0.63	0.90
PFOA			
Maximum	1.90	1.48	1.90
Minimum	0.51	0.38	0.38
Median	0.93	0.61	0.80

Raw data were adopted from Hsu *et al.*, 2013.

* Converted by $DP = CP \times KP \times Vd$.

DP: Dietary intakes of PFOS and PFOA (ng $kg\text{-}bw^{-1}$ day^{-1}).

CP: PFOS and PFOA concentrations in serum (ng mL^{-1}). (Table S5)

KP: Elimination rates of PFOS and PFOA (day^{-1}).

Vd: Distribution volumes of PFOS and PFOA (mL $kg\text{-}bw^{-1}$)

Fig. S1.

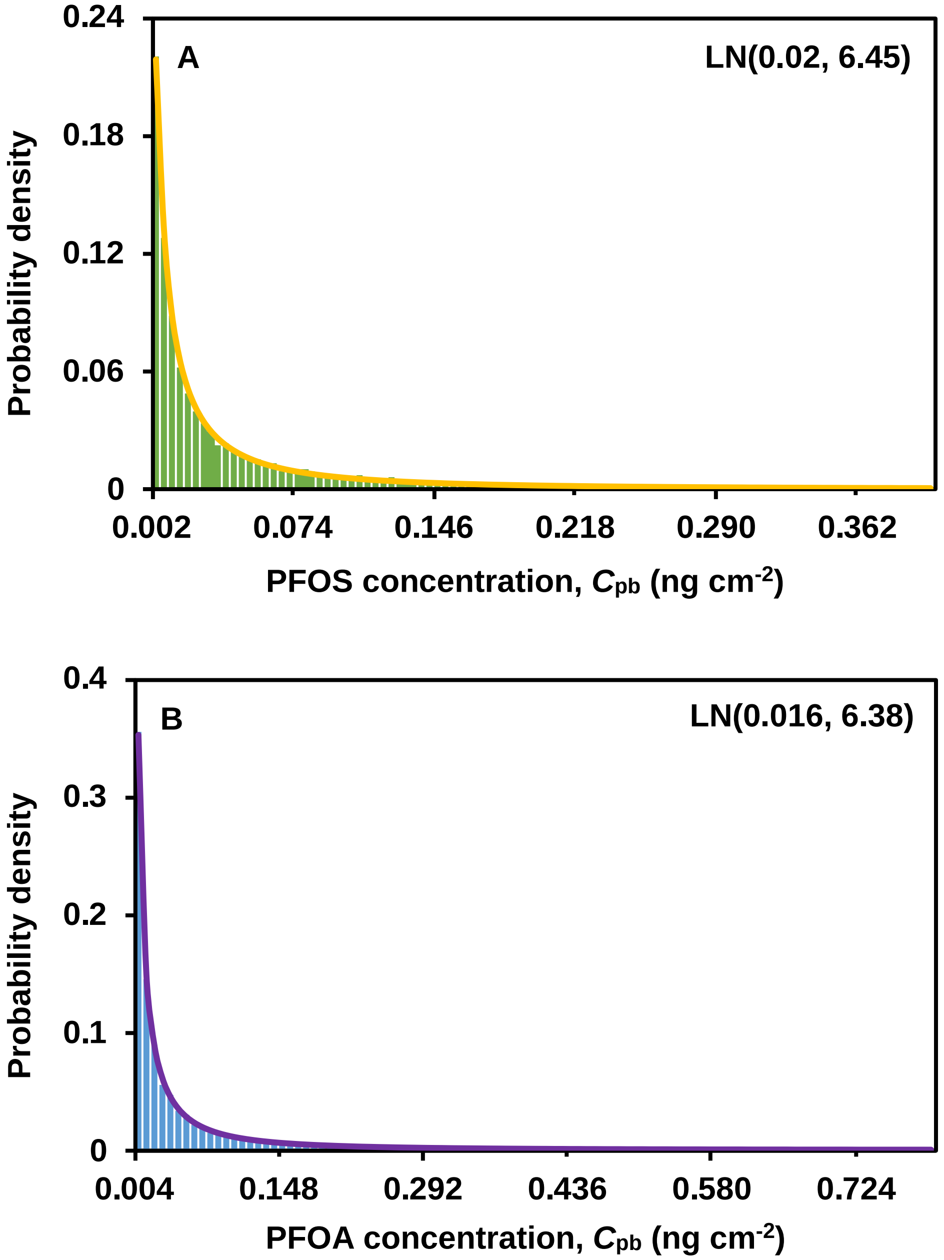

A
LN(0.02, 6.45)
Probability density
0.24
0.18
0.12
0.06
0
0.002
0.074
0.146
0.218
0.290
0.362
PFOS concentration, Cpb (ng cm-2)
B
LN(0.016, 6.38)
Probability density
0.4
0.3
0.2
0.1
0
0.004
0.148
0.292
0.436
0.580
0.724
PFOA concentration, Cpb (ng cm-2)

Fig. S2.

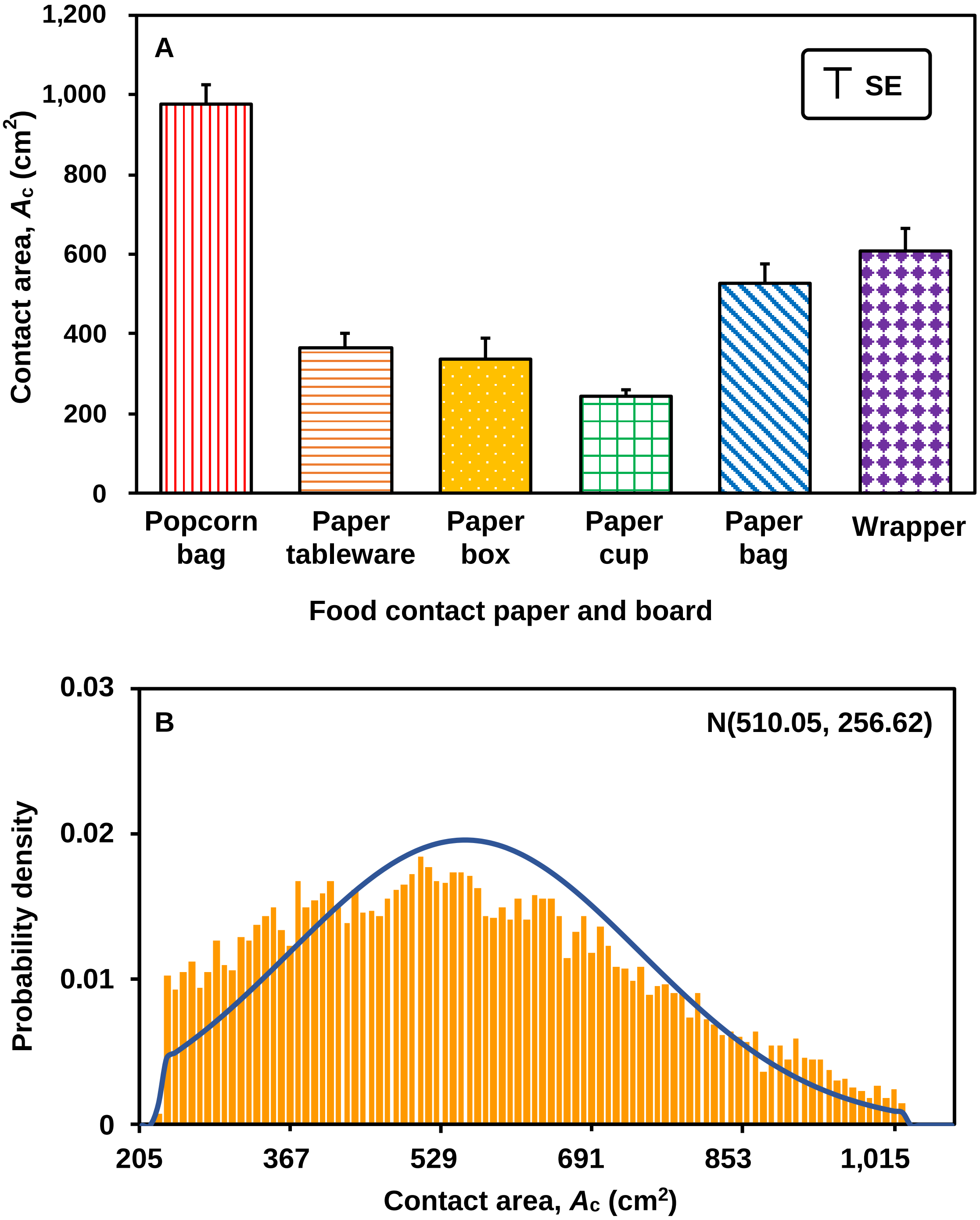

A
SE
1,200
1,000
800
600
400
200
0
Contact area, Ac (cm2)
Popcorn bag
Paper tableware
Paper box
Paper cup
Paper bag
Wrapper
Food contact paper and board
B
N(510.05, 256.62)
0.03
0.02
0.01
0
Probability density
205
367
529
691
853
1,015
Contact area, Ac (cm2)

Fig. S3.

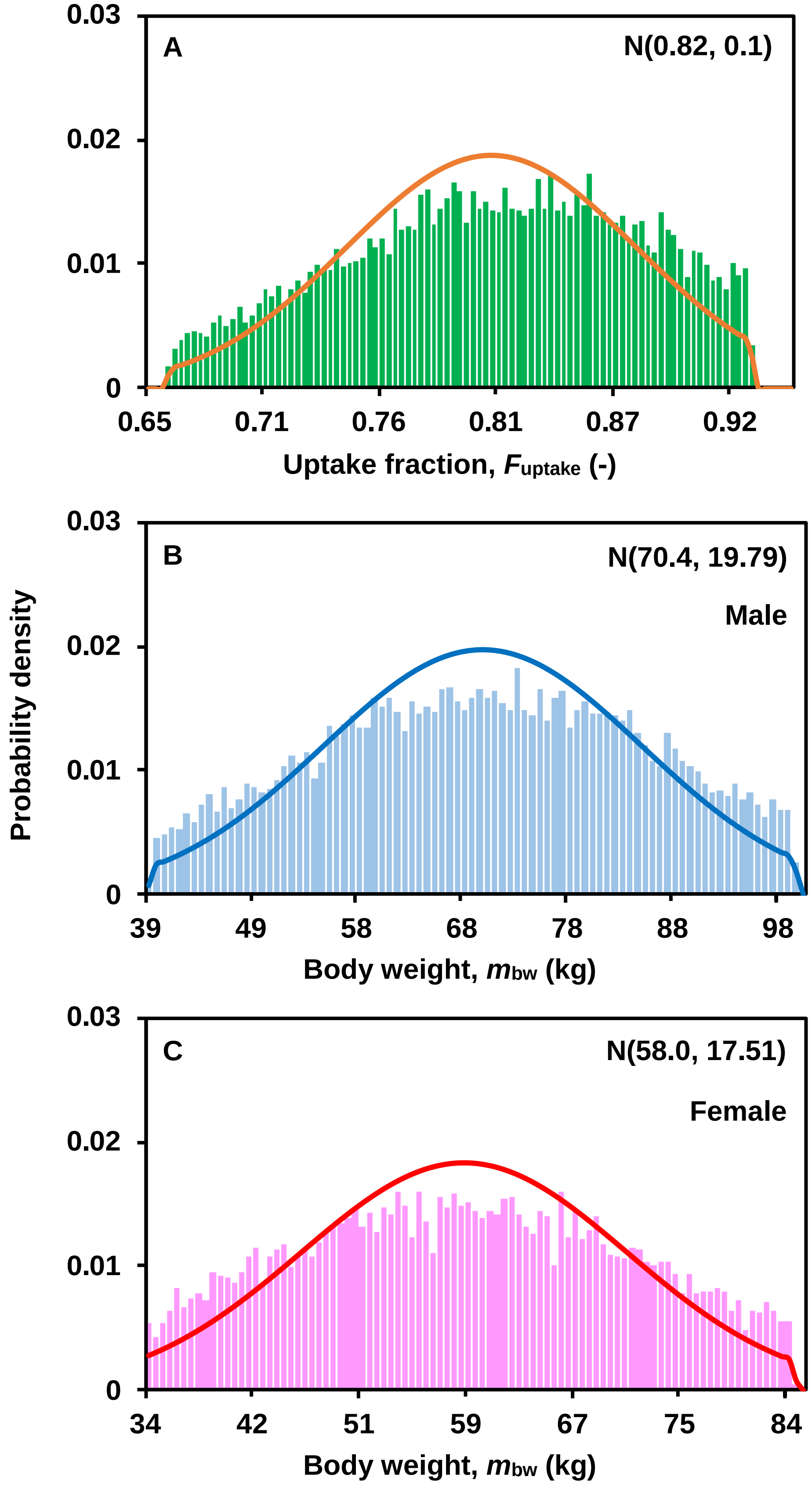

A
N(0.82, 0.1)
0.03
0.02
0.01
0
0.65
0.71
0.76
0.81
0.87
0.92
Uptake fraction, F_{uptake} (-)
Probability density
B
N(70.4, 19.79)
Male
0.03
0.02
0.01
0
39
49
58
68
78
88
98
Body weight, m_{bw} (kg)
C
N(58.0, 17.51)
Female
0.03
0.02
0.01
0
34
42
51
59
67
75
84
Body weight, m_{bw} (kg)

Fig. S4.

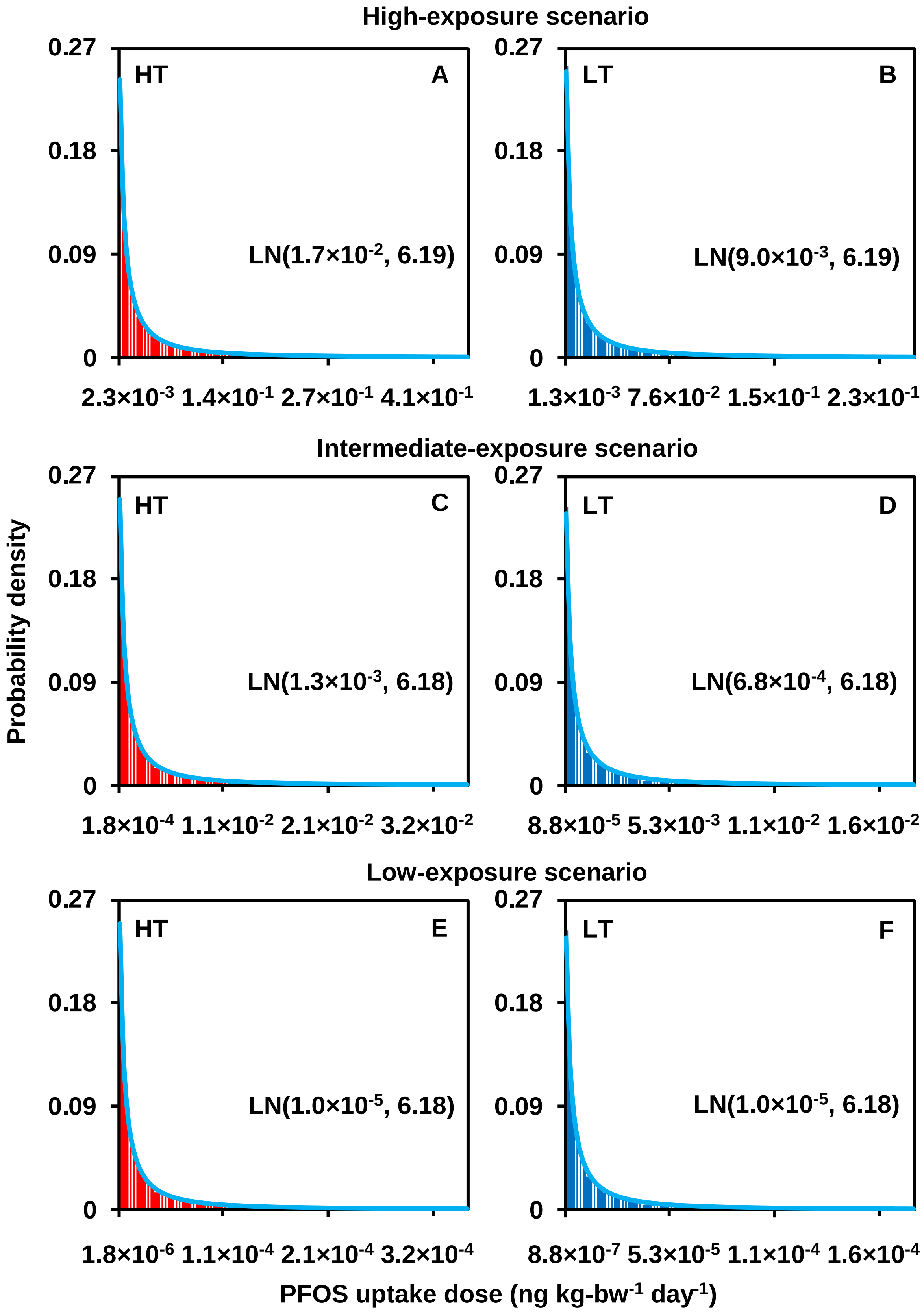
High-exposure scenario
HT
A
LN(1.7×10-2, 6.19)
LT
B
LN(9.0×10-3, 6.19)
2.3×10-3 1.4×10-1 2.7×10-1 4.1×10-1
1.3×10-3 7.6×10-2 1.5×10-1 2.3×10-1
Intermediate-exposure scenario
HT
C
LN(1.3×10-3, 6.18)
LT
D
LN(6.8×10-4, 6.18)
1.8×10-4 1.1×10-2 2.1×10-2 3.2×10-2
8.8×10-5 5.3×10-3 1.1×10-2 1.6×10-2
Low-exposure scenario
HT
E
LN(1.0×10-5, 6.18)
LT
F
LN(1.0×10-5, 6.18)
1.8×10-6 1.1×10-4 2.1×10-4 3.2×10-4
8.8×10-7 5.3×10-5 1.1×10-4 1.6×10-4
Probability density
0.27
0.18
0.09
0
PFOS uptake dose (ng kg-bw-1 day-1)

Fig. S5.

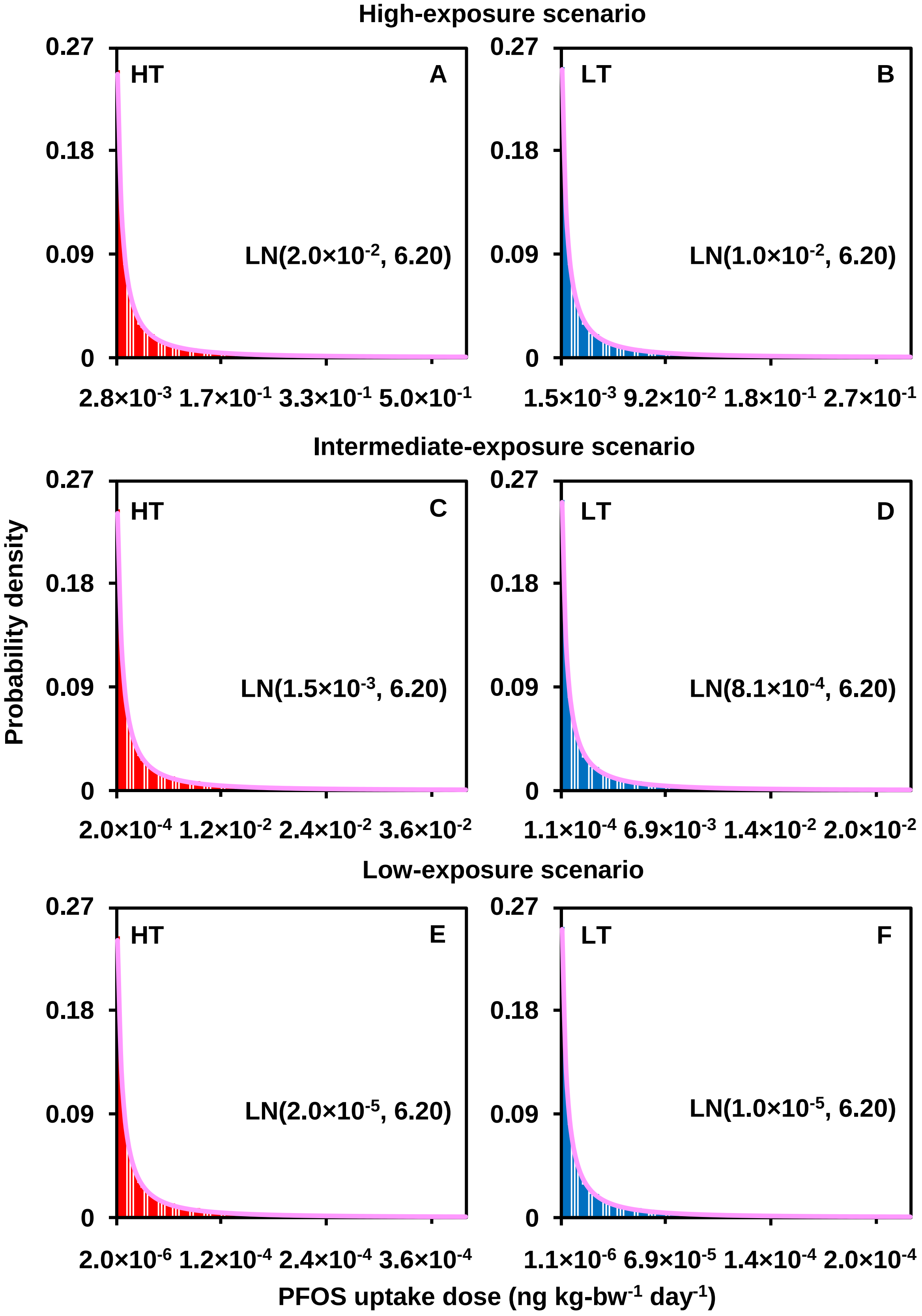
High-exposure scenario
HT
A
LN(2.0×10-2, 6.20)
2.8×10-3 1.7×10-1 3.3×10-1 5.0×10-1
LT
B
LN(1.0×10-2, 6.20)
1.5×10-3 9.2×10-2 1.8×10-1 2.7×10-1
Intermediate-exposure scenario
HT
C
LN(1.5×10-3, 6.20)
2.0×10-4 1.2×10-2 2.4×10-2 3.6×10-2
LT
D
LN(8.1×10-4, 6.20)
1.1×10-4 6.9×10-3 1.4×10-2 2.0×10-2
Low-exposure scenario
HT
E
LN(2.0×10-5, 6.20)
2.0×10-6 1.2×10-4 2.4×10-4 3.6×10-4
LT
F
LN(1.0×10-5, 6.20)
1.1×10-6 6.9×10-5 1.4×10-4 2.0×10-4
0.27
0.18
0.09
0
Probability density
PFOS uptake dose (ng kg-bw-1 day-1)

Fig. S6.

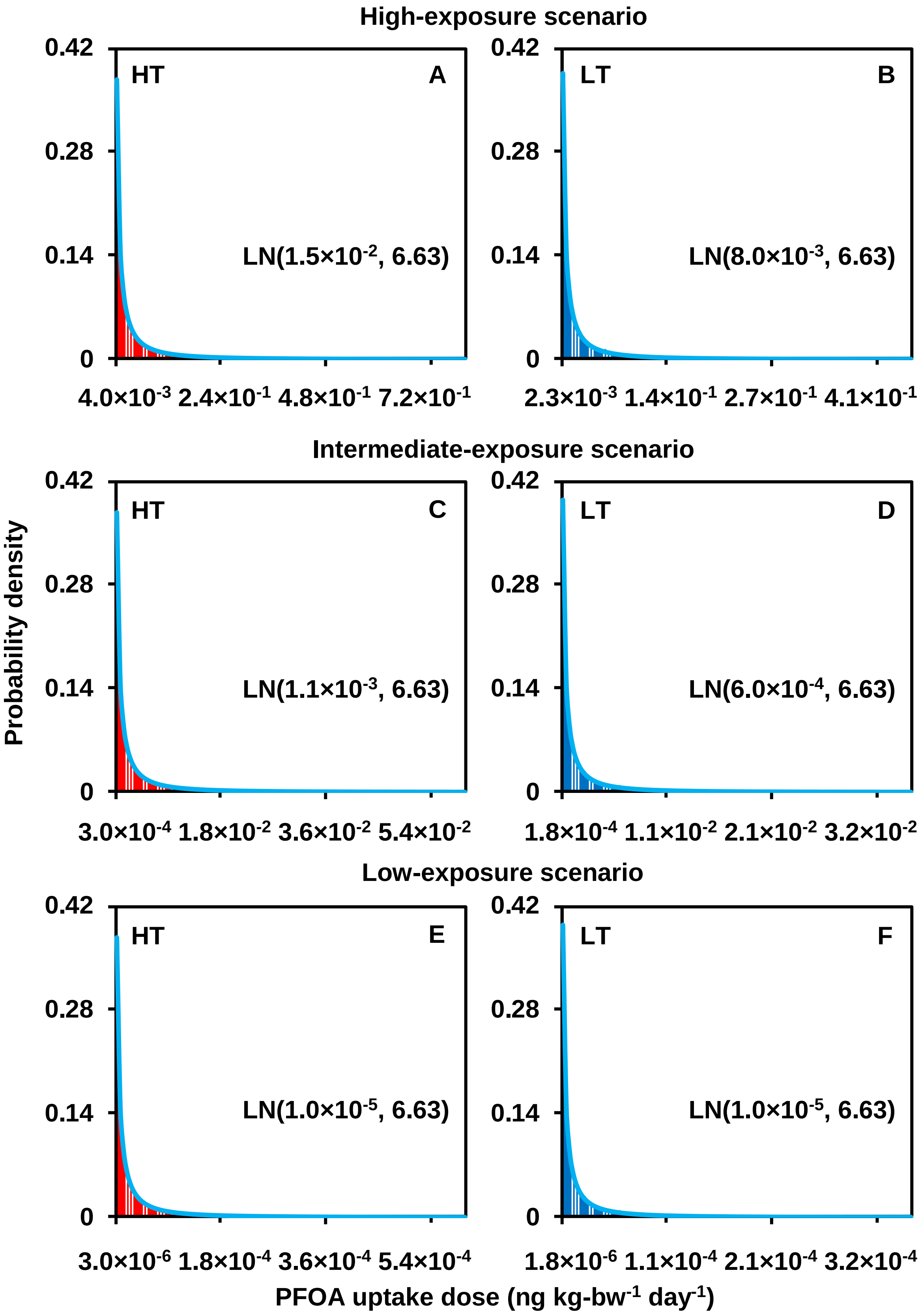
High-exposure scenario
0.42
0.28
0.14
0
HT
A
LN(1.5×10-2, 6.63)
4.0×10-3 2.4×10-1 4.8×10-1 7.2×10-1
LT
B
LN(8.0×10-3, 6.63)
2.3×10-3 1.4×10-1 2.7×10-1 4.1×10-1
Intermediate-exposure scenario
HT
C
LN(1.1×10-3, 6.63)
3.0×10-4 1.8×10-2 3.6×10-2 5.4×10-2
LT
D
LN(6.0×10-4, 6.63)
1.8×10-4 1.1×10-2 2.1×10-2 3.2×10-2
Low-exposure scenario
HT
E
LN(1.0×10-5, 6.63)
3.0×10-6 1.8×10-4 3.6×10-4 5.4×10-4
LT
F
LN(1.0×10-5, 6.63)
1.8×10-6 1.1×10-4 2.1×10-4 3.2×10-4
Probability density
PFOA uptake dose (ng kg-bw-1 day-1)

Fig. S7.

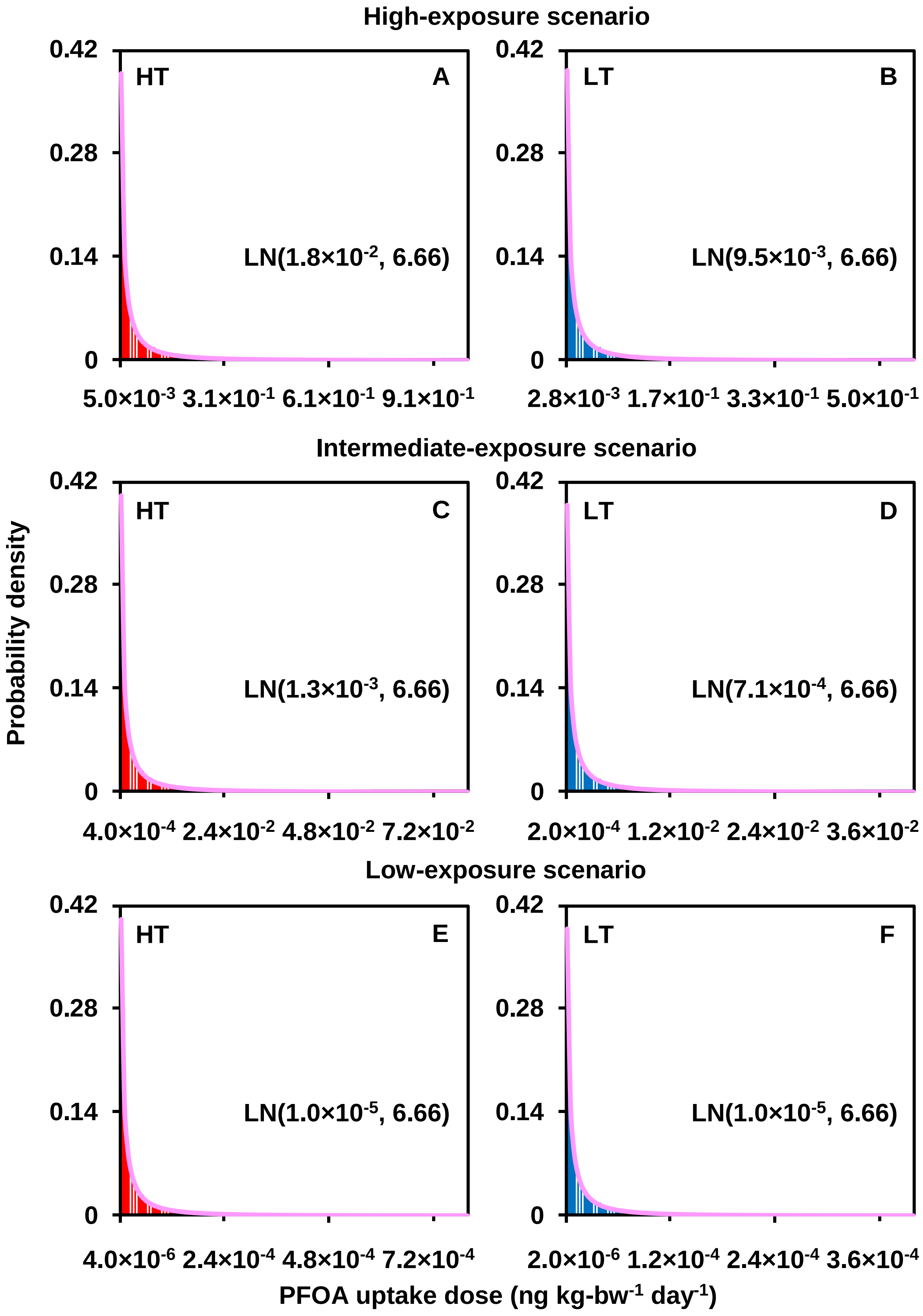
High-exposure scenario
HT
A
LN(1.8×10-2, 6.66)
LT
B
LN(9.5×10-3, 6.66)
5.0×10-3 3.1×10-1 6.1×10-1 9.1×10-1
2.8×10-3 1.7×10-1 3.3×10-1 5.0×10-1
Intermediate-exposure scenario
HT
C
LN(1.3×10-3, 6.66)
LT
D
LN(7.1×10-4, 6.66)
4.0×10-4 2.4×10-2 4.8×10-2 7.2×10-2
2.0×10-4 1.2×10-2 2.4×10-2 3.6×10-2
Low-exposure scenario
HT
E
LN(1.0×10-5, 6.66)
LT
F
LN(1.0×10-5, 6.66)
4.0×10-6 2.4×10-4 4.8×10-4 7.2×10-4
2.0×10-6 1.2×10-4 2.4×10-4 3.6×10-4
0.42
0.28
0.14
0
Probability density
PFOA uptake dose (ng kg-bw-1 day-1)
